# Strong health systems are learning health systems

**DOI:** 10.1371/journal.pgph.0000229

**Published:** 2022-03-16

**Authors:** Kabir Sheikh, Seye Abimbola

**Affiliations:** 1 Alliance for Health Policy and Systems Research, World Health Organization, Geneva, Switzerland; 2 School of Public Health, University of Sydney, NSW, Australia; PLOS: Public Library of Science, UNITED STATES

## Health system investments: Great expectations

The COVID-19 pandemic has brought to the surface discussions about the need for stronger health systems, especially in poorer countries. Global health funders are beginning to back up their rhetoric in this area with investments. The EU, USA, UK, France, and Germany, among others are investing billions [1] on the health systems strengthening (HSS) agenda in poorer countries. Among multilateral funders, the Global Fund currently spends $1 billion each year [2] on their “resilient and sustainable systems for health” portfolio, and Gavi’s new 5-year strategy will likely infuse $1.7 billion [3] into strengthening health and immunization systems. These sums are insignificant compared to the loss to lives and livelihoods and the trillions [4] in economic losses that are the cost of inaction—and this slew of investments in HSS is welcome news.

But there is a hitch. Policymakers and practitioners who work in those health systems are accustomed to seeing the global interest in HSS fluctuate. It is mainly when emergencies (such as Ebola or COVID-19) strike, that the world wakes up to the importance of HSS, and by then, it is often too late [5]. We expect a lot from our health systems. We expect them to deliver high quality, efficient care, and provide universal coverage. We also expect them to be resilient in the face of shocks, keep us safe, and shield us from crises. When faced with catastrophe, we ask health systems to deliver results quickly.

There is still a disconnect between our lofty expectations from health systems, and the inputs that they receive to make them stronger. Health systems do not become stronger overnight. Many investments purport to strengthen them but fail to do so. The gains of well-intentioned and well-designed investments to strengthen health systems are limited, when they do not focus on strengthening the ability of health systems to learn.

## Learning investments are neglected

Learning–i.e., the ability to create, gather and use relevant knowledge and intelligence, to bring about improvements in performance–is a key neglected element in global HSS efforts. Learning investments help health systems manage change better–by being better prepared for future challenges, and by driving cultures of analysis, self-critique, and innovation; ultimately leading to greater self-reliance [6].

**Fig 1:**
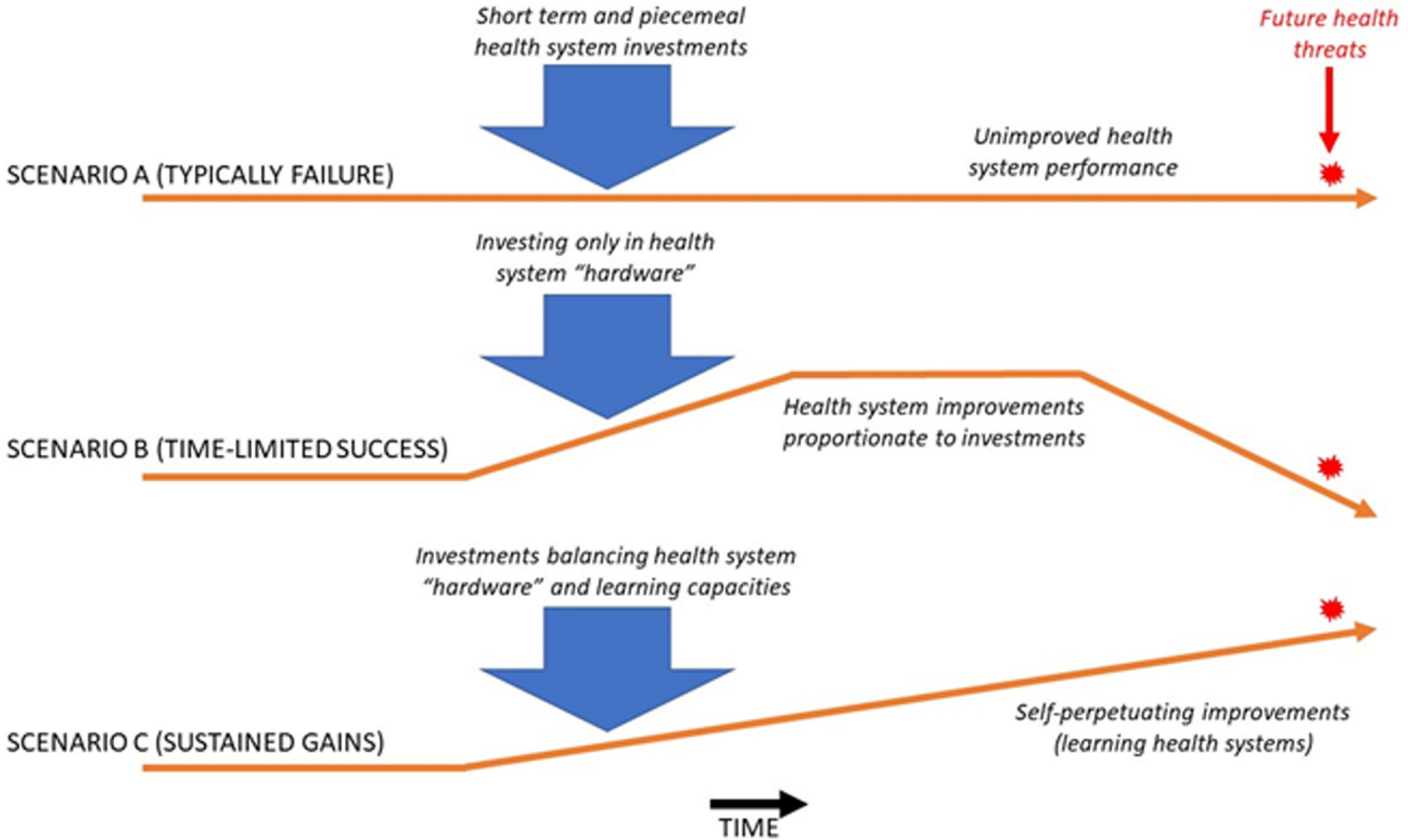
Outcomes of different types of investments in health systems.

However, instances of purposeful investment in learning health systems are regrettably rare. Operational exigencies in health systems tend to crowd out the “softer” work of learning, which is often regarded as having less immediate or predictable benefits. Learning remains a neglected focus of most health systems and is poorly resourced compared to other types of HSS investments [7]. Many HSS investments are short term [8] and fragmented (scenario A in **Fig 1**) and are often ineffective. Another common type of investment (scenario B in **Fig 1**) is focused largely on strengthening the “hardware” of health systems [9]–for example medical products, supply chains and infrastructure. These investments are necessary but limited in their potential for lasting change, since their benefits can erode in the absence of health systems’ capacity to sustain and adapt such hardware to changing needs and contexts.

What is needed then? A report produced by the WHO-hosted Alliance for Health Policy and Systems Research [10] describes what is needed to build Learning Health Systems [11]. Learning health systems institutionalize key learning functions such as data-driven decision support, health systems research, community feedback, solution-sharing, innovation labs, technology assessment, and specialized intelligence functions such as disaster forecasting or behavior change analysis. Learning health systems also effectively create and deploy the human capacities needed to perform these learning functions. Ultimately learning health systems are more likely to sustain and accelerate improvements in performance through their ability to continuously innovate and adapt to change (scenario C in **Fig 1**).

## Three examples of learning investments that made a difference

First, it is vital for all countries, especially poorer countries, to *strengthen domestic capacities for health system intelligence*–including strategic use of ecological, epidemiological, demographic, and behavioral data; and crucially, health systems research and policy analysis. Commencing in the 1970s, Thailand’s preparation for UHC reforms included a strong commitment on investing in domestic capacity for health systems research and policy analysis [12]. Experts were trained over several decades and employed in policy institutes closely affiliated to the health ministry. Thailand has since achieved a high level of financial protection and improved equity in access to health services, thanks in part to the shaping and steering role of these technical experts.

Second, health systems in poorer countries need to do more to *recognize and make use of innovations emerging from day-to-day experience*. Health systems are rich repositories of such tacit and experiential learning, but this is seldom identified or codified in ways that can be shared widely or scaled up [13]. One example from South Africa [14] is how ART (anti-retroviral therapy) clubs–initially a small-scale NGO-led innovation to improve adherence to HIV/AIDS treatment–became institutionalized as government policy. The Western Cape province recognized, piloted, and eventually scaled up ART clubs as part of the public antiretroviral therapy programme–a measure that has contributed to the expansion of the ART programme–and ultimately to the lowering of mortality rates [15] of patients on ART.

Third, health systems must *listen to and learn from the communities they serve*, for whom stronger health systems have the most tangible benefits. Following the Republic of Korea’s [11] experience with the MERS outbreak in 2015 during which lack of transparency was recognized to have marred public trust in government agencies, the government consulted with citizen groups. They consequently introduced new laws for pandemic preparedness, mandating citizens’ rights to information about public health strategies and requiring employers to compensate people in treatment or quarantine because of outbreaks. These provisions resulted in greater awareness and participation of citizens in the COVID-19 response and helped create high levels of trust in public health measures–the engine of Korea’s successful response in the early phases of the pandemic.

## Culture eats strategy

Learning investments are not “quick fixes”. They do not guarantee rapid or predictable returns. They require the patience and perseverance to nurture human capital for learning, and to conceive and grow learning institutions. This is the price of developing stronger health systems–unlike a lot of current investments that are short-term or focus exclusively on developing the hardware of health systems, the gains from learning investments are likely to be long-lasting and genuinely transformational.

“Culture eats strategy for breakfast” is a famous management quote. The best laid plans to strengthen health systems and prevent future epidemics risk eventual failure if they are not accompanied by investments in the people and institutions that drive a learning culture in health systems.
